# Calculation of absolute binding free energies between the hERG channel and structurally diverse drugs

**DOI:** 10.1038/s41598-019-53120-6

**Published:** 2019-11-12

**Authors:** Tatsuki Negami, Mitsugu Araki, Yasushi Okuno, Tohru Terada

**Affiliations:** 1 Graduate School of Agricultural and Life Sciences, The University of Tokyo, 1-1-1 Yayoi, Bunkyo-ku, Tokyo, 113-8657 Japan; 2 Graduate School of Medicine, Kyoto University, 53 Shogoin-Kawaharacho, Sakyo-ku, Kyoto, 606-8507 Japan; 3 Interfaculty Initiative in Information Studies, The University of Tokyo, 7-3-1 Hongo, Bunkyo-ku, Tokyo, 113-0033 Japan

**Keywords:** Virtual drug screening, Computational biophysics, Molecular modelling

## Abstract

The human *ether-a-go-go*-related gene (hERG) encodes a voltage-gated potassium channel that plays an essential role in the repolarization of action potentials in cardiac muscle. However, various drugs can block the ion current by binding to the hERG channel, resulting in potentially lethal cardiac arrhythmia. Accordingly, *in silico* studies are necessary to clarify the mechanisms of how these drugs bind to the hERG channel. Here, we used the experimental structure of the hERG channel, determined by cryo-electron microscopy, to perform docking simulations to predict the complex structures that occur between the hERG channel and structurally diverse drugs. The absolute binding free energies for the models were calculated using the MP-CAFEE method; calculated values were well correlated with experimental ones. By applying the regression equation obtained here, the affinity of a drug for the hERG channel can be accurately predicted from the calculated value of the absolute binding free energy.

## Introduction

The human *ether-a-go-go*-related gene (hERG) encodes a voltage-gated potassium (Kv) channel that generates a rapid delayed rectifier K^+^ current (*I*_Kr_) across the cell membrane of cardiac myocytes in an action potential-dependent manner, a process considered critical for the repolarization of the action potential^1–3^. Impaired functioning of the hERG channel, including from various mutations, can cause prolongation of the QT interval as observed by electrocardiogram (ECG), potentially leading to lethal cardiac arrhythmia^2,3^. QT prolongation can also be caused by the blockade of the hERG channel by certain drugs^4,5^. Since structurally diverse drugs are known to cause this serious side effect, all drug candidates are required to be tested for hERG blocking activities during preclinical development. However, tests using *in vitro* and *in vivo* experiments are very costly and time-consuming. Therefore, the development of *in silico* tools for predicting the binding affinities of drugs for the hERG channel is expected to reduce the cost and improve the efficiency of the drug development process.

Numerous studies have attempted to clarify the structural mechanisms underlying hERG-drug interactions using both experimental and/or computational methods. Based on its sequence similarity to other Kv channels, of which the structures have been determined (for review, see ref.^3^), it has been suggested that the hERG channel has a tetrameric structure, with each subunit containing six transmembrane helices (S1–S6). In this structure, helices S5 and S6 form the ion-conducting pore domain, while helices S1–S4 form the voltage sensor domain (VSD). Drugs are believed to enter the pore of the hERG channel from the cytoplasmic side. Previous mutagenesis studies have shown that the affinities of various hERG blockers are decreased by mutations of residues T623, S624, and V625, near the cytoplasmic end of the selectivity filter, and Y652 and F656, located in helix S6^6–15^. In particular, docking simulations using homology models of the hERG channel have suggested that residues S624, Y652, F656, and F557 directly interact with various drugs^10–17^.

Several methods have been developed for predicting the affinity of a drug to the hERG channel^18^, such as the recent report by Chemi *et al*.^19^ of a method that used the three-dimensional quantitative structure–activity relationship for the prediction. Ogura *et al*.^20^ used a support vector machine to predict the hERG inhibitory activity of a drug. However, the accuracy of these methods depends on the amount of experimental data included in the training data set, and the predicted affinities or inhibitory activities may not be accurate for drugs that differ structurally from those in the training data set. Boukharta *et al*.^21^ applied an MD-based method to predict the affinities of drugs to the hERG channel. This involved predicting the structure of the complex formed between each drug and the hERG channel and then performing MD simulations for the complex and for the drug alone to estimate the binding free energy. This estimate used the linear interaction energy method, which is based on an empirical relationship between the intermolecular interaction energies and the binding free energy. However, Boukharta *et al*. only predicted the affinities of structurally related drugs. In addition, they used a homology model for the structure of the hERG channel, which may have limited the accuracy of the prediction.

The experimental structure of the hERG channel had long remained unsolved, despite its biological and pharmaceutical importance. Recently, however, the structures of hERG and its closely related homolog, rat Eag1 (rEag1), were solved by cryo-electron microscopy (cryo-EM)^22,23^. Similar to other Kv channels, hERG and rEag1 channels have a tetrameric structure with a four-fold rotational symmetry. The pore-forming regions (helices S5–S6) of the four subunits assemble to form the pore domain, which has an ion-conducting pore at the center along the symmetry axis, with the VSDs (helices S1–S4) surrounding. However, cryo-EM structures revealed a domain arrangement unique to the hERG/rEag1 channel. Indeed, while the other Kv channels have a domain-swapped arrangement where the VSD of a given subunit is located in proximity to the pore-forming region of the neighboring subunit, the VSD of the hERG/rEag1 channel is located in proximity to its own pore-forming region. Consistent with this arrangement, the linker sequence of the hERG/rEag1 channel connecting the VSD with the pore-forming region is shorter than those of the other Kv channels. In addition, the rEag1 channel is in a closed conformation with a closed pore^22,23^, whereas the hERG channel is in an open conformation with the pore expanded on the cytoplasmic side of the selectivity filter, forming a large cavity termed the central cavity^22^. Residues that have been suggested to be involved in drug binding are located around the central cavity^22^, implying that drugs bind to this cavity. Furthermore, the structure of the central cavity of the hERG channel appears to be different from those of the other Kv channels with open pores^22^.

Using the cryo-EM structure of the hERG channel, in this study, we predicted the complex structures that occur between the hERG channel and structurally diverse drugs using docking simulations. In addition, we calculated the absolute binding free energies for selected drugs using a molecular-dynamics (MD)-based method termed Massively Parallel Computation of Absolute binding Free Energy with well-Equilibrated states (MP-CAFEE)^24,25^. We then examined the interactions between the drugs and the channel and evaluated the correlation between the calculated binding free energy values and those determined experimentally. Finally, we present a method for predicting the affinity of a drug for the hERG channel based on these correlations.

## Results and Discussion

### Stability of the pore domain structure in MD simulations

**Figure 1:**
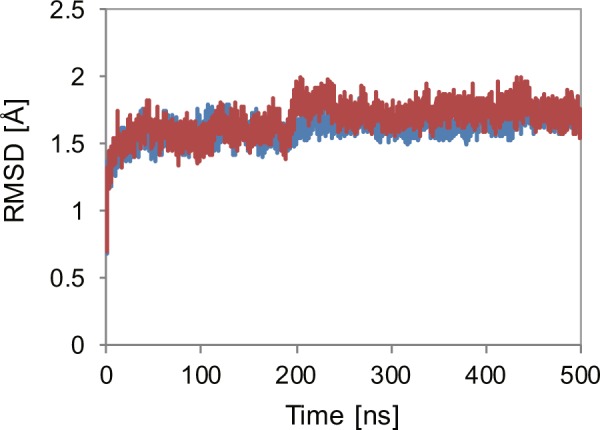
Time evolutions of the Cα RMSDs from the initial structure calculated for the transmembrane regions of the pore domain (residues 547–575 and 607–666) during the MD simulations for the VSD–pore–C-linker system (blue) and the pore-domain-only system (red).

We first examined the stability of the pore domain structure of the hERG channel using MD simulations. To this end, we compared the stability between the two systems: the VSD–Pore–C-linker system where the VSD–Pore–C-linker part of the hERG channel (residues 398–736) is embedded in a solvated lipid bilayer and the pore-domain-only system where the pore domain (residues 544–671) is embedded in a solvated lipid bilayer. The Cα RMSDs from the initial structures calculated for the transmembrane regions of the pore domain (residues 547–575 and 607–666) were less than 2 Å throughout the 500-ns simulations for each system (Fig. 1). These results indicated that the structure of the pore domain is stably maintained without its flanking domains (the VSD and the C-linker). Thus, for computational efficiency, we used the pore-domain-only system for further analysis.

### hERG-drug interactions predicted by docking simulations

**Figure 2:**
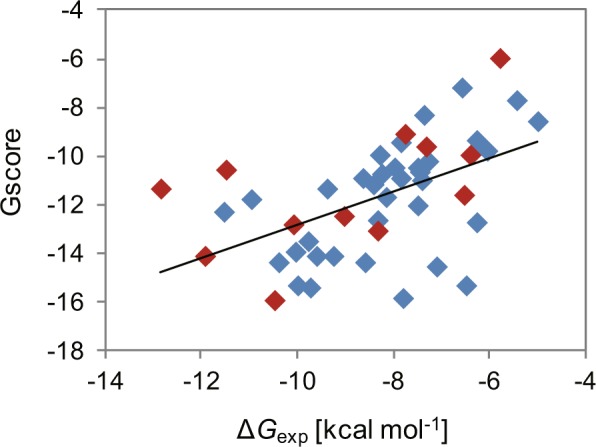
Plot of the Glide scores (Gscore) of the top-ranked poses against the experimental values of the binding free energy (Δ*G*_exp_) for the 47 compounds. Data points colored red represent the results of the 12 drugs for which the absolute binding free energies were calculated with the MP-CAFEE method. The solid line represents the regression line calculated from all the data with a coefficient of determination (*R*^2^) of 0.264.

Docking simulations of the pore domain of the hERG channel were performed for 47 drugs (Supplementary Table S1), of which inhibition constants have been determined using the same experimental method^26,27^. As described above, S624, Y652, and F656 appear to be involved in the interactions with these drugs. Therefore, we set the center of the search space as the center of the mass of the Y652 residues of the four subunits, which are located between S624 and F656 in the tertiary structure. Docking simulations were performed with the Glide module of the Schrödinger Suite by following its induced-fit docking (IFD) protocol configured to allow conformational changes of these residues. Figure 2 shows a plot of the Glide docking scores (Gscores) of the top-ranked poses against the experimental binding free energies. These were weakly correlated with a coefficient of determination (*R*^2^) of 0.264.

We next examined the interactions of the top-ranked poses with the key binding residues (S624, Y652, and F656) of the channel for 15 drugs, of which interactions have been investigated using mutation experiments (Table 1). A residue was considered to be in contact with the ligand if a non-hydrogen sidechain atom of the residue was within 4.0 Å from a non-hydrogen atom of the ligand. Y652 and F656 formed contacts with all the docking poses examined here. However, previous experiments have indicated that the mutations of Y652 and F656 did not affect the affinities of bepridil or moxifloxacin, respectively^8,9^. In addition, S624 formed contacts with all ligands, except haloperidol and ranolazine. Previous experiments have shown that mutations of S624 significantly reduce the affinities of clofilium, ibutilide, and E-4031, which is consistent with our docking results. However, the affinity of sotalol was not affected by the mutation of S624.

**Table 1:** Contacts between the top-ranked poses and key binding residues (S624, Y652, and F656), and experimental results of key-residue mutants.

Compound	S624		Y652		F656		Ref. of exp. data
	Docking^a^	Exp.^b^	Docking^a^	Exp.^b^	Docking^a^	Exp.^b^	
Clofilium	+	+	+	+	+	+	Perry *et al*.^13,14^
Astemizole	+		+	+	+		Saxena *et al*.^17^
Ibutilide	+	+	+	+*	+	+*	Perry *et al*.^13^
Dofetilide	+		+	+*	+	+*	Kamiya *et al*.^9^
E-4031	+	+*	+	+*	+	+*	Kamiya *et al*.^9^
Cisapride	+		+	+	+	+	Mitcheson *et al*.^15^
Terfenadine	+		+	+	+	+	Mitcheson *et al*.^15^
Haloperidol	−		+	+	+		Saxena *et al*.^17^
Bepridil	+		+	−	+	+*	Kamiya *et al*.^9^
Amiodarone	+	+	+	+	+	+	Saxena *et al*.^17^
Propafenone	+		+		+	+	Witchel *et al*.^11^
Quinidine	+		+		+	+	Sanchez-Chapula *et al*.^7^
Ranolazine	−		+	+	+	+	Du *et al*.^10^
Sotalol	+	−	+		+		Zhang *et al*.^30^
Moxifloxacin	+		+		+	−	Alexandrou *et al*.^8^

^a^Contacts with the top-ranked pose. A residue was considered to be in contact with a ligand if a non-hydrogen sidechain atom of the residue was within 4.0 Å from a non-hydrogen atom of the ligand.

^b^Experimental results of the mutations. + indicates that a large contribution to the ligand binding is suggested (>10-fold change in IC50). * is added when the IC_50_ values were estimated from the data of the fractional block by using the Hill equation (fractional block = [L]^*h*^ /(IC_50_ + [L]^*h*^)), assuming *h* = 1. – indicates that no contribution to binding affinity is suggested in the reference.

**Figure 3:**
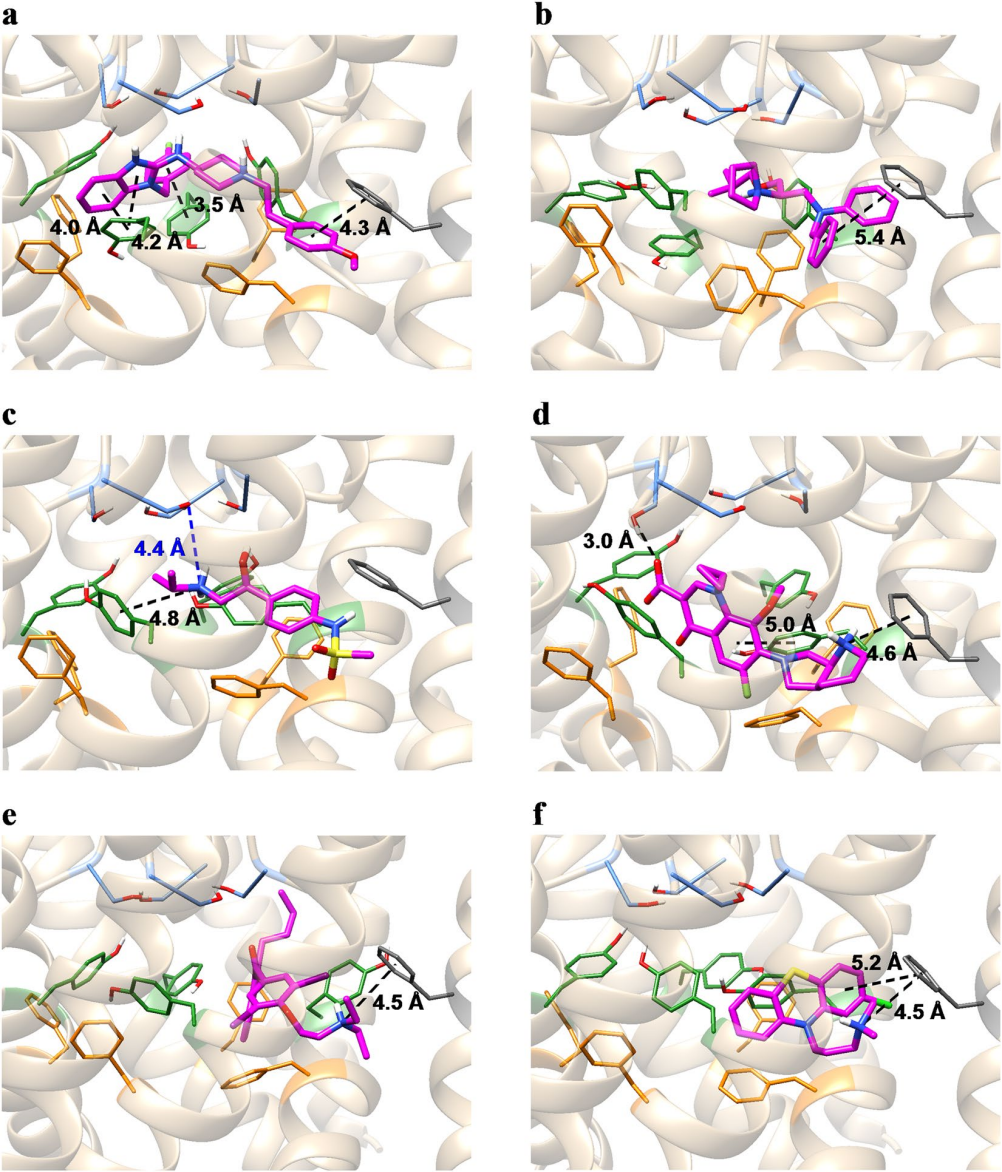
Top-ranked poses of astemizole (**a**), bepridil (**b**), sotalol (**c**), moxifloxacin (**d**), amiodarone (**e**), and chlorpromazine (**f**). Protein backbone structures are shown in ribbon form. Ligand molecules and the sidechains of residues S624, Y652, F656, and F557 are shown in stick representation (magenta: ligand molecules; light blue: S624; green: Y652; orange: F656; dark gray: F557). The black dashed lines represent hydrogen bonds, π-π interactions, or cation-π interactions as identified by the Maestro module of the Schrödinger Suite. The blue dashed line in (**c**) represents the distance between the nitrogen atom of the amine of sotalol and the closest oxygen atom of the hydroxyl group of S624. All molecular graphic images were produced using UCSF Chimera^63^.

To evaluate the importance of the apparent discrepancies between the results of the contact analysis and those determined experimentally, we further inspected the interactions of these drugs with the channel (Fig. 3). First, we analyzed the docking pose of astemizole, which had no discrepancy with the experimental results. Astemizole was located in the center of the pore and surrounded by the key binding residues, S624, Y652, and F656 (Fig. 3a). The aromatic rings of astemizole were located close to those of three Y652 residues of the four subunits, suggesting a favorable contribution of π-π interactions^28,29^ to ligand binding. In addition, we found contacts with F557, which has recently been suggested to contribute to astemizole binding^17^. As described above, the docking pose of bepridil formed contacts with Y652, whereas the mutation of Y652 does not affect the affinity of bepridil^9^. Furthermore, the docking pose of bepridil indicated that there are no π-π interactions with Y652 (Fig. 3b), suggesting that Y652 does not have a large contribution to bepridil binding. Therefore, the docking pose of bepridil is consistent with the experimental results. Figure 3c shows the docking pose of sotalol. Although a methyl group of the *N*-isopropyl group of sotalol is within 4.0 Å from the Cβ atom of S624, the distance between the amine group of sotalol and the hydroxy group of S624 is 4.4 Å, indicating that sotalol does not form a hydrogen bond with S624. Therefore, this pose seems to be consistent with the experimental results that S624 does not have a large contribution to sotalol binding^30^. Another study has suggested that F656 does not have a large contribution to moxifloxacin binding^8^. The docking pose of moxifloxacin indicated that it interacts more strongly with Y652 (through π-π interactions) than F656. Therefore, the mutation of F656 is not likely to greatly affect the affinity of moxifloxacin for the channel.

**Figure 4:**
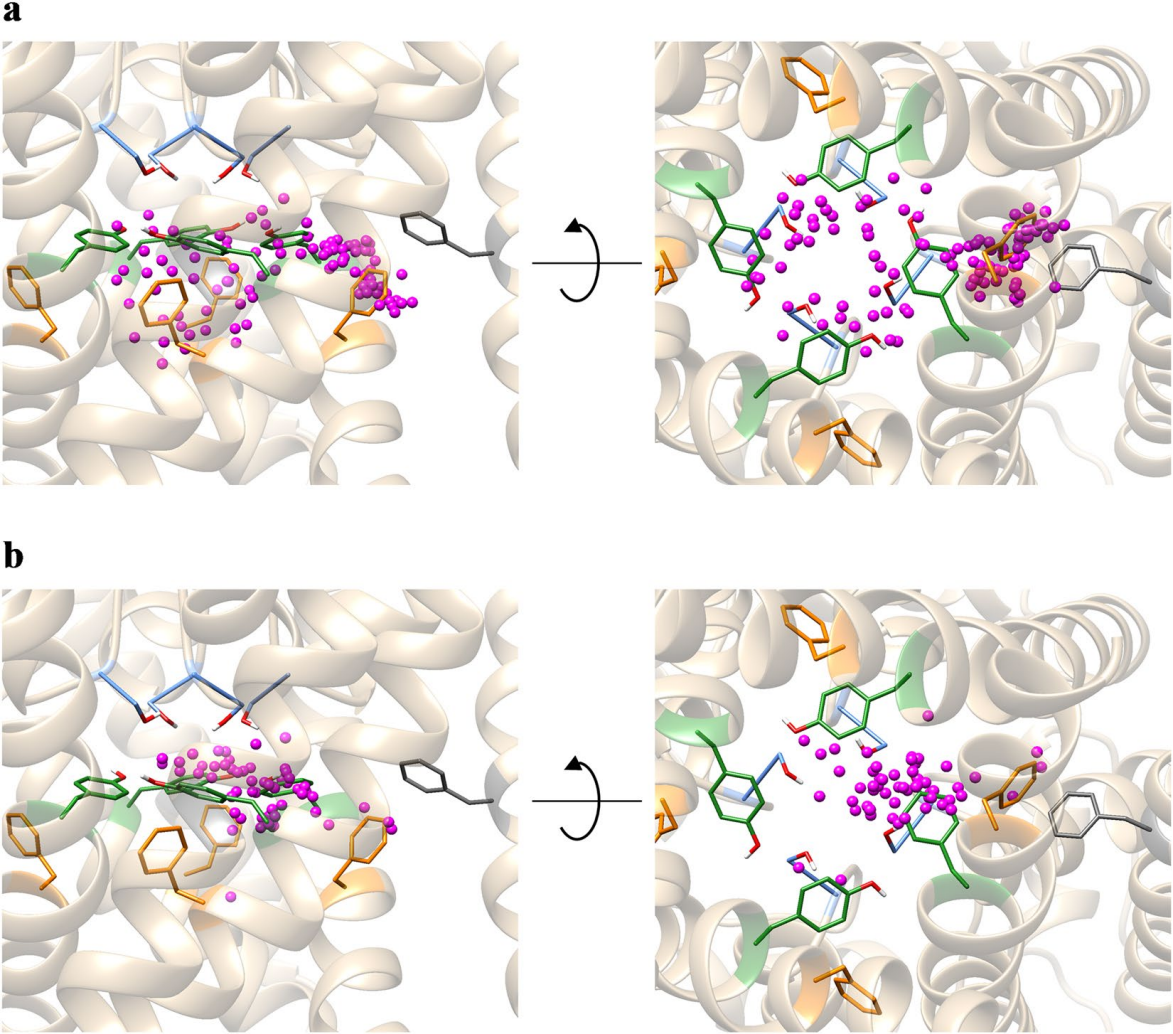
Distributions of the aromatic rings (**a**) and positively charged amines (**b**) in all the top-ranked poses. The nitrogen atoms of the amines and the geometric centers of the aromatic rings of the ligand molecules are shown as magenta balls. Protein backbone structures are shown in ribbon form. The sidechains of residues S624, Y652, F656, and F557 are shown in stick representations and colored light blue, green, orange, and dark gray, respectively. 90°-rotated views are shown on the right panels.

We next analyzed the spatial distributions of the aromatic rings and positively charged amines of all 47 drugs, since most of the drugs have these groups (Fig. 4). The geometric centers of the aromatic rings were near Y652 or F656, demonstrating the importance of π-π interactions for ligand binding (Fig. 4a). The distribution of the nitrogen atoms of the positively charged amines indicated that most of them were located near S624 or Y652. In addition, they appeared to form hydrogen bonds with the hydroxyl group of S624, or cation-π interactions with the aromatic ring of Y652, indicating a large contribution to ligand binding (Fig. 4b). However, three drugs, amiodarone, moxifloxacin, and chlorpromazine, were located near F557. Their docking poses are shown in Fig. 3d–f, respectively. Since the distances between the nitrogen atoms of the positively charged amines of these drugs and the center of mass of the aromatic ring of F557 are about 4.5 Å, they appear to form cation-π interactions with the aromatic ring of F557.

Overall, the docking poses were considered reasonable because the ligands interacted with the key residues as proposed in the experimental studies. However, the correlation (*R*^2^ = 0.264) between the docking scores (Gscores) and the experimental values of the binding free energies was not considered sufficient. Therefore, we next performed MD simulations for selected drugs to more accurately calculate the absolute binding free energies.

### Absolute binding free energies

**Figure 5:**
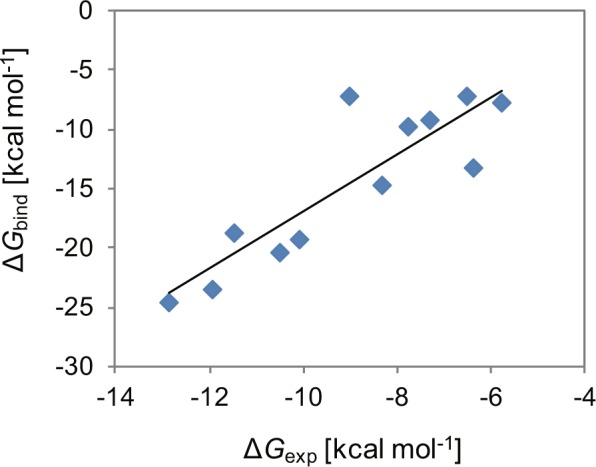
Plot of the calculated values of the absolute binding free energy (Δ*G*_bind_) against those determined experimentally (Δ*G*_exp_) for the 12 compounds. The solid line represents the regression line with a coefficient of determination (*R*^2^) of 0.767.

We calculated the absolute binding free energies of the drugs using the MP-CAFEE method^24^. Because this method is based on MD simulation and is computationally expensive, we selected 12 drugs (Supplementary Fig. S1), paying attention to the diversities in their molecular structures and in the experimental binding free energy values (−5.77 – −12.84 kcal mol^−1^). The resulting complex structures from the previous docking simulations were used as the initial structures for the MD simulations. The calculated absolute binding free-energy values are listed in Supplementary Table S2 and are plotted against those determined experimentally in Fig. 5. The coefficient of determination (*R*^2^) was 0.767, indicating that the correlation between the calculated and experimental values was greatly improved by calculating the absolute binding free energy.

Clofilium had the lowest experimental binding free energy (i.e., the strongest affinity for the channel) among the 12 drugs. Although the Gscore for clofilium was middle-ranked among the other drugs, its calculated absolute binding free-energy value was the lowest and in agreement with the experimental result. Thus, the MP-CAFEE method, which is based on rigorous theory, can evaluate the absolute binding free energy more accurately than empirical scoring functions.

**Figure 6:**
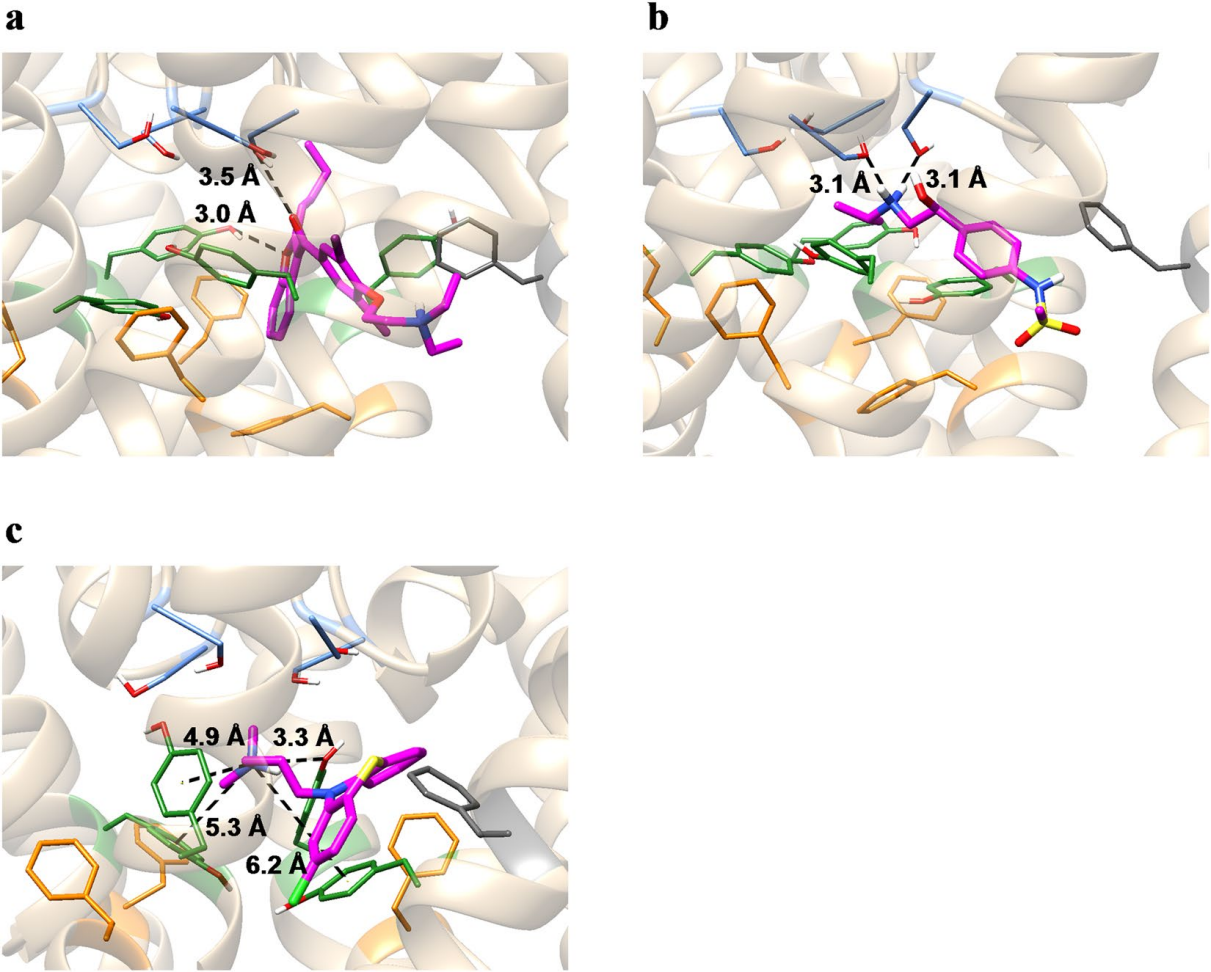
Equilibrium structures of amiodarone (**a**), sotalol (**b**), and chlorpromazine (**c**). Ligand molecules and the sidechains of residues S624, Y652, F656, and F557 are shown in stick representation (magenta: ligand molecules; light blue: S624; green: Y652; orange: F656; dark gray: F557). The black dashed lines represent hydrogen bonds, π-π interactions, or cation-π interactions as identified by the Maestro module of the Schrödinger Suite.

**Figure 7:**
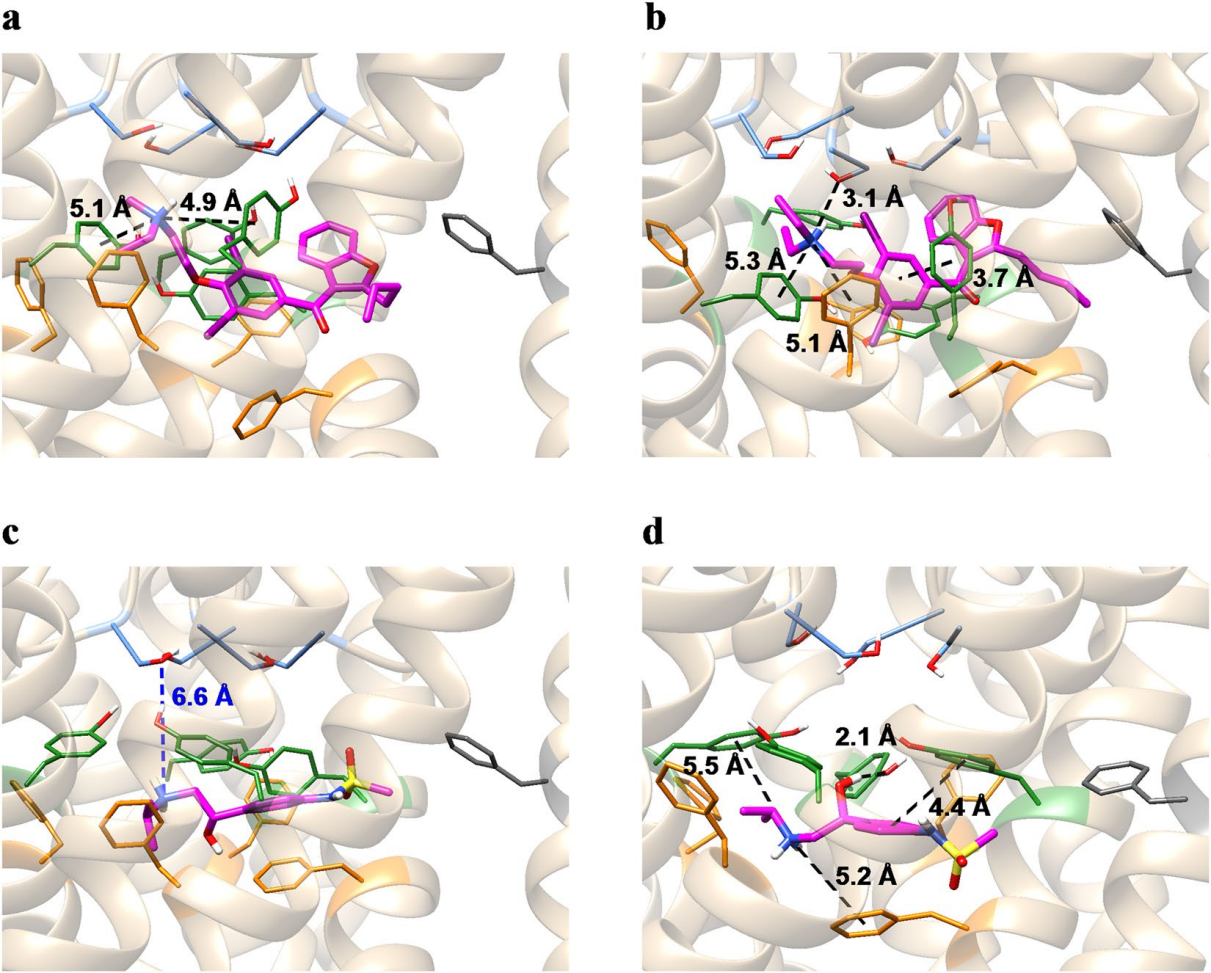
Top-ranked poses of the additional docking simulations and their equilibrium structures. (**a**) Docking pose of amiodarone. (**b**) Equilibrium structure of amiodarone. (**c**) Docking pose of sotalol. (**d**) Equilibrium structure of sotalol. Ligand molecules and the sidechains of residues S624, Y652, F656, and F557 are shown in stick representation (magenta: ligand molecules; light blue: S624; green: Y652; orange: F656; dark gray: F557). The black dashed lines represent hydrogen bonds, π-π interactions, or cation-π interactions as identified by the Maestro module of the Schrödinger Suite. The blue dashed line in (**c**) represents the distance between the nitrogen atom of the amine of sotalol and the closest oxygen atom of the hydroxyl group of S624.

However, there were two drugs for which the calculated values largely deviated from the regression line. One was amiodarone, in which the positively charged amine exceptionally formed a cation-π interaction with F557 (see Fig. 3e). Its affinity was greatly underestimated in our calculation. In contrast, the deviation from the regression line for the calculated value of another exception, chlorpromazine (see Fig. 3f), was considered small. In this study, the initial structure of the free-energy calculation was taken from an equilibrium ensemble generated by multiple runs of the MD simulation. In the equilibrium structure, the positively charged amine of amiodarone was detached from F557 and did not form cation-π interactions with any aromatic residues (Fig. 6a). Conversely, chlorpromazine changed its binding conformation to form a cation-π interaction with the aromatic ring of Y652, along with a hydrogen bond with the hydroxyl group of another Y652 (Fig. 6c). Since the positively charged amine formed a hydrogen bond with S624, or a cation-π interaction with Y652, the docking pose of amiodarone with such interactions may have a lower binding free energy. Because such a docking pose was not included in the results produced by the Glide module using the default output settings, we increased the number of outputs of the initial docking to 50, and repeated the docking simulation for amiodarone. We found that the top-ranked pose formed a cation-π interaction with the aromatic rings of Y652 (Fig. 7a). In addition, it formed contacts with F656, which is in agreement with experimental results^17^. During the MD simulations, the binding conformation of amiodarone slightly changed. In the equilibrium structure, the positively charged amine formed a hydrogen bond with S624, along with a weak cation-π interaction with Y652 (Fig. 7b). In addition, the absolute binding free energy calculated with the MP-CAFEE method was −16.70 kcal mol^−1^, which was 9.63 kcal mol^−1^ lower than the previous value determined.

Another drug that exhibited a large deviation from the regression line was sotalol. The affinity of sotalol was overestimated. The equilibrium structure indicated that the positively charged amine formed hydrogen bonds with the S624 residues of two subunits (Fig. 6b). Since the experimental results indicated that S624 is not involved in the binding of sotalol, the docking pose was likely incorrect. To obtain docking poses that were more consistent with the experimental results, we performed a docking simulation for sotalol, applying an excluded volume penalty to the ligand that comes close to S624. Figure 7c,d show the top-ranked pose and the equilibrium structure of sotalol. In both structures, sotalol did not interact with S624. In addition, the calculated absolute binding free energy value was −11.79 kcal mol^−1^, which was closer to the regression line by 1.36 kcal mol^−1^ than the previous value.

**Figure 8:**
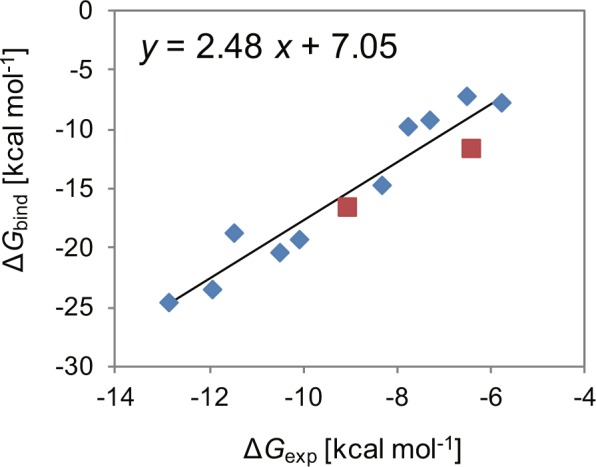
Plot of the calculated values of the absolute binding free energy (Δ*G*_bind_) against those determined experimentally (Δ*G*_exp_) for the 12 compounds after additional calculations for amiodarone and sotalol. Data points colored red represent the results of the additional calculations for amiodarone and sotalol. The solid line represents a regression line with a coefficient of determination (*R*^2^) of 0.909. The regression equation is shown above the graph, in which *x* is Δ*G*_exp_ and *y* is Δ*G*_bind_.

Figure 8 shows a plot of the calculated absolute binding free energy values against those determined experimentally after the corrections for amiodarone and sotalol. The coefficient of determination (*R*^2^) was 0.909, a value further improved from our previous result. The binding free energy of a new drug can be accurately predicted using the regression equation (shown in Fig. 8). First, the drug is docked into the central cavity of the pore domain of the hERG channel. The top-ranked pose is then used in subsequent absolute binding free energy calculations. If the interactions between the drug and the hERG channel were also studied experimentally, the consistency between the docking pose and the experimental results should be examined carefully. If the drug has aromatic rings, they likely interact with Y652 and F656. It should be kept in mind that positively charged amines interact with S624 or Y652 in most cases. Second, the absolute binding free energy is calculated using the MP-CAFEE method. Third, the regression equation is applied to the calculated value to evaluate the predicted binding free-energy value that corresponds to the experimental value.

Finally, we evaluated the accuracy of our method using data for four further drugs as a test set. These drugs were not included among the 12 drugs used to calculate the regression equation and their structures (Supplementary Fig. S2) differed from those of the 12 drugs (Supplementary Fig. S1). When the calculated absolute binding free energy values were plotted against the experimental values, the points representing the four additional drugs lay close to the regression line (Supplementary Fig. S3a). The calculated values were converted to predicted binding free energy values by applying the regression equation. The root mean squared error (RMSE) between the predicted and the experimental values was 0.73 kcal mol^−1^ (Supplementary Table S3). For comparison, we calculated the QPlogHERG descriptors for the original 12 drugs and the additional four drugs using the QikProp module of the Schrödinger Suite^31^ (Supplementary Table S3). The QPlogHERG descriptor predicts the common logarithm of the IC_50_ value for blockage of the hERG channel. The calculation failed for clofilium; therefore, the QPlogHERG values of the other 11 drugs were plotted against their experimental binding free energy values (Δ*G*_exp_) and the regression equation was determined (Supplementary Fig. S3b). The coefficient of determination (*R*^2^) was 0.765, which is comparable to that calculated from the initial values of the absolute binding free energy (Fig. 5) but worse than that calculated from the values after the corrections for amiodarone and sotalol (Fig. 8). The QPlogHERG values calculated for the four drugs were converted to binding free energy values by applying the regression equation. The RMSE between the predicted and experimental values was 1.40 kcal mol^−1^. These results confirmed that our method could accurately predict the affinity of a drug to the hERG channel.

It should be noted that in this study we used the cryo-EM structure of the hERG channel in the open conformation. The hERG channel can adopt multiple conformations depending on its particular state, including the active, inactive, and closed states. The structure of the central cavity of the pore domain, to which drugs can bind, also varies between the states. Therefore, it may be difficult to accurately predict the affinity of a drug that binds to the channel in a different conformation from the open conformation. In fact, a recent study has suggested that the cryo-EM structure of the hERG channel used in this study may not be suitable for predicting the affinity of cavalli-2^32^. Nonetheless, the accuracy of the binding free-energy prediction can be further improved if the structures of the hERG channel in other conformations are determined.

## Methods

### Initial structure preparation

The coordinates of the cryo-EM structure of the hERG channel were obtained from the Protein Data Bank (PDB) (PDB ID: 5VA2)^22^. The structures of the disordered extracellular loops (residues 578–582 and 598–602) were modeled using Modeller v9.18^33^. Potassium ions were placed at the S1 and S3 sites of the selectivity filter and water molecules were placed at the S0, S2, and S4 sites^22^. The protonation states of ionizable residues at pH 7 were determined using the Protein Preparation Wizard of the Schrödinger Suites^34^.

Experimental inhibition constants (*K*_i_) have been experimentally determined by [H^3^]-dofetilide displacement assays for 47 structurally diverse drugs^26,27^ (Supplementary Table S1). The structures of these drugs were obtained from the PubChem database^35^. The protonation state at pH 7 was determined for each drug and the structure was optimized with the OPLS-3 force field^36^. These calculations were conducted using the LigPrep module of the Schrödinger Suites^37^.

### Docking simulations

We performed docking simulations with the Glide module of the Schrödinger Suite, following the induced-fit docking (IFD) protocol^38^. The pore domain (residues 544–671) extracted from the tetrameric channel structure was used as the receptor. In the IFD protocol, docking simulations were performed as three steps: initial docking, receptor refinement, and redocking. In the initial docking step, residues S624, Y652, and F656 were mutated to alanine residues and the van der Waals radii of both the ligand and protein atoms were scaled down to 70%. The center of the box that defines the search space was then set as the center of mass of the Y652 residues of the four subunits. Initial docking was performed using Glide’s standard precision (SP) mode^39,40^ and 20 poses were generated for each ligand. In the subsequent receptor refinement step, residues mutated in the initial docking step were restored and the structures of the residues within 5.0 Å of the docked ligand were refined with the OPLS-3 force field by using the Prime module^41,42^. In the final redocking step, the ligand was docked again to the refined protein structure after removal of the ligand from the complex of the previous step using Glide’s extra precision (XP) mode^43^. Finally, the generated poses were ranked using the IFD score.

### MD simulations

In this study, we performed MD simulations for both the ligand-free and ligand-bound structures of the hERG channel in a solvated lipid bilayer environment. Water molecules were placed in the central cavity of the channel before the channel structure was embedded in the lipid bilayer using the CHARMM-GUI server^44,45^. Two systems with different protein lengths were constructed. The first was the VSD–pore–C-linker system, which was composed of a part of the hERG channel encompassing the VSDs, the pore domain, and the C-linkers, about 600 1-palmitoyl-2-oleoyl-*sn*-glycero-3-phosphocholine (POPC) molecules, and 62,000 water molecules. The second system was a pore-domain-only system, which was composed of the pore domain of the hERG channel, 290 POPC molecules, and 18,000 water molecules. MD simulations were also performed for ligand-only systems, each of which was composed of one ligand molecule and approximately 6,700 water molecules. K^+^ and Cl^−^ ions were added to each system so that the net charge of the whole system was zero and the salt concentration was approximately 0.15 M. The CHARMM36 force field was used for the proteins^46^, ions^47^, and POPC^48^. The TIP3P model^49^ was used for water. The parameters for the drug compounds were determined with the CHARMM General Force Field^50^ (CGenFF) by using the CHARMM-GUI ligand modeler^51^. Each system was energy-minimized and equilibrated following the CHARMM-GUI protocol. MD simulations to generate equilibrium ensembles (production runs) were performed under the constant-*NPT* condition. The temperature was maintained at 303.15 K using a Nose-Hoover thermostat^52,53^. The pressure was maintained at 1 bar with the Parrinello-Rahman barostat^54^. Electrostatic interactions were calculated using the particle mesh Ewald (PME) method^55^ with a real space cutoff of 1.2 nm. Van der Waals interactions were calculated with a modified Lennard-Jones potential, where the force was smoothly switched to zero between 1.0 and 1.2 nm. The lengths of the bonds involving the hydrogen atoms were constrained with the LINCS algorithm to allow for the use of a time step of 2 fs^56^. Production runs for the ligand-free hERG channels and ligand-only systems were performed for 500 ns and 20 ns, respectively. For the channel-drug complexes, 50-ns production runs were performed five times with different initial velocities. All MD simulations were performed with Gromacs 4.6^57^.

### Calculation of absolute binding free energies

Absolute protein-ligand binding free energies were calculated for the top-ranked channel-drug complex models with the MP-CAFEE method^24^. Calculations were performed following the protocols reported in previous studies^24,58,59^. In the MP-CAFEE method, a well-equilibrated structure is required as the initial structure of the free energy calculation. To obtain such structures, 50-ns production runs were performed five times with different initial velocities for each channel-drug complex. Then, the moving average of the intermolecular interaction energy (the sum of the van der Waals and the electrostatic interaction energies) between the protein and the ligand was calculated with a window size of 2 ns. The snapshot with the lowest interaction energy was then selected as the initial structure of the free energy calculation. As for the ligand-only systems, a 20-ns production run was performed once for each ligand and the final structure was used as the initial structure. The free energy differences between the λ = 0 state, where the ligand fully interacts with its surroundings, and the λ = 1 state, where the intermolecular interactions between the ligand and the others are completely uncoupled, were calculated for the protein-ligand complexes (Δ*G*_comp_) and the ligand-only systems (Δ*G*_lig_). Here, 32 intermediate states were considered between the λ = 0 and the λ = 1 states to make the adjacent two states close enough, between which the free-energy difference could be calculated. MD simulations for the free-energy calculation were performed six times with different initial velocities. The length of the MD simulation for each state was 2 ns for the protein-ligand complex and 1.2 ns for the ligand-only system. The temperature was maintained at 303.15 K using a Nose-Hoover thermostat and the pressure was maintained at 1 bar using a Berendsen barostat^60^. The free energy difference was calculated using the Bennett acceptance ratio method^61,62^. The absolute binding free energy (Δ*G*_bind_) was calculated as Δ*G*_bind_ = Δ*G*_comp_ − Δ*G*_lig_. The MD simulations were performed on the K computer. The 2-ns MD simulation for the protein–ligand complex using eight nodes (64 cores) took approximately 30 h. The 1.2-ns MD simulation for the ligand-only system using 1.5 nodes (12 cores) also took approximately 30 h. These calculations were performed six times for each of the 32 intermediate states. Thus, the total computational cost was 54,720 node-hours. Because the calculations were independent, they were executed in parallel.

## Supplementary information

Supplementary Information

## Data Availability

All data generated or analyzed during this study are included in this published article and its Supplementary Information files.
