# Supplementary material for: Calculation of absolute binding free energies between the hERG channel and structurally diverse drugs
Source: Sci Rep. 2019 Nov 12;9:16586. doi: 10.1038/s41598-019-53120-6 (PMC6851376; doi:10.1038/s41598-019-53120-6)
Supplement: Supplementary file 1 — Supplementary Information [file 41598_2019_53120_MOESM1_ESM.pdf]

Supplementary Information

Calculation of absolute binding free energies between the hERG channel and structurally diverse drugs

Tatsuki Negami, Mitsugu Araki, Yasushi Okuno, and Tohru Terada

Supplementary Table S1. The 47 compounds for which the docking simulations were performed and their experimental inhibition constants (K_i) and binding free energies (ΔG_{exp}).

Compound	SMILES	K_i [nM]	ΔG_{exp} [kcal mol^{-1}][a]	Ref.
Amiodarone	c1cccc(c12)oc(CCCC)c2C(=O)c3cc(I)c(c(I)c3)OCC[NH+](CC)CC	308	−9.031	Yu et al.[1]
Aripiprazole	C1CC(=O)Nc(c12)cc(cc2)OCCCC[NH+](CC3)CCN3c(c4Cl)cccc4Cl	2450	−7.782	Diaz et al.[2]
Astemizole	COc(cc1)ccc1CC[N@H+](CC2)CC[C@@H]2Nc([nH+]c(c34)cccc3)n4Cc5ccc(F)cc5	2.5	−11.931	Yu et al.
Bepridil	CC(C)COC[C@H]([NH+]1CCCC1)CN(c2ccccc2)Cc3ccccc3	170	−9.389	Diaz et al.
Chlorpromazine	C[NH+](C)CCCN1c(cccc2)c2Sc(c13)ccc(Cl)c3	2518	−7.766	Yu et al.
Cisapride	c1c(Cl)c(N)cc(OC)c1C(=O)N[C@@H]2[C@H](OC)C[N@@H+](CC2)CCCOc3ccc(F)cc3	54	−10.080	Yu et al.
Clofilium	CCCCCCC[N+](CC)(CC)CCCCc1ccc(Cl)cc1	0.55	−12.843	Diaz et al.
Clozapine	C1C[NH+](C)CCN1C2=Nc(cc(Cl)cc3)c3Nc(c24)cccc4	1200	−8.212	Diaz et al.
Desipramine	C[NH2+]CCCN(c(c12)cccc1)c3c(CC2)cccc3	5530	−7.292	Diaz et al.
Diltiazem	C[NH+](C)CCN1C(=O)[C@H](OC(=O)C)[C@H](c2ccc(cc2)OC)Sc(c13)cccc3	31200	−6.250	Diaz et al.
Diphenhydramine	c1ccccc1C(OCC[NH+](C)C)c2ccccc2	4140	−7.466	Yu et al.

Dofetilide	CS(=O)(=O)Nc(cc1)ccc1CC[N@@H+](C)CCOc(cc2)ccc2NS(=O)(=O)C	5.4	−11.467	Diaz et al.
Domperidone	c1cccc(c12)[nH]c(=O)n2CCC[N@H+](CC3)CC[C@@H]3n4c(=O)[nH]c(c45)cc(Cl)cc5	220	−9.234	Diaz et al.
Droperidol	c1cc(F)ccc1C(=O)CCCN(CC2)CC=C2n3c(=O)[nH]c(c34)cccc4	120	−9.599	Yu et al.
E-4031	CS(=O)(=O)Nc(cc1)ccc1C(=O)[C@H]2CC[N@@H+](CC2)CCc3cccc(n3)C	13	−10.938	Diaz et al.
Ebastine	CC(C)(C)c(cc1)ccc1C(=O)CCC[N@@H+](CC2)CC[C@@H]2OC(c3ccccc3)c4ccccc4	100	−9.709	Diaz et al.
Fexofenadine	[O-]C(=O)C(C)(C)c1ccc(cc1)[C@@H](O)CCC[N@H+](CC2)CC[C@@H]2C(O)(c3ccccc3)c4ccccc4	32000	−6.234	Diaz et al.
Flecainide	[NH2+]1CCCC[C@@H]1CNC(=O)c2c(OCC(F)(F)F)ccc(c2)OCC(F)(F)F	4540	−7.411	Diaz et al.
Fluoxetine	FC(F)(F)c1ccc(cc1)O[C@H](CC[NH2+]C)c2ccccc2	2230	−7.839	Diaz et al.
Gatifloxacin	[O-]C(=O)c(c1=O)cn(C2CC2)c(c13)c(OC)c(c(F)c3)N(C[C@H]4C)CC[NH2+]4	127900	−5.400	Diaz et al.
Grepafloxacin	[O-]C(=O)c(c1=O)cn(C2CC2)c(c13)cc(c(F)c3C)N(C[C@H]4C)CC[NH2+]4	68900	−5.772	Diaz et al.
Haloperidol	c1cc(F)ccc1C(=O)CCC[N@H+](CC2)CC[C@]2(O)c3ccc(Cl)cc3	90	−9.772	Diaz et al.
Ibutilide	CS(=O)(=O)Nc(cc1)ccc1[C@H](O)CCC[N@H+](CC)CCCCCCC	5.1	−11.501	Diaz et al.

Imipramine	C[NH+](C)CCCN(c(c12)cccc1)c3c(CC2)cccc3	4480	−7.419	Diaz et al.
Ketoconazole	CC(=O)N1CCN(CC1)c2ccc(cc2)OC[C@H](CO3)O[C@]3(Cn4cc[nH+]c4)c5c(Cl)cc(Cl)cc5	19500	−6.533	Diaz et al.
Mesoridazine	C[N@@H+]1CCCC[C@H]1CCN2c(cccc3)c3Sc(c24)ccc(c4)[S@](=O)C	1790	−7.971	Diaz et al.
Mibefradil	c1cc(F)cc(c12)CC[C@]([C@H]2C(C)C)(OC(=O)COC)CC[N@@H+](C)CCCc(n3)[nH]c(c34)cccc4	660	−8.572	Diaz et al.
Moxifloxacin	[O-]C(=O)c(c1=O)cn(C2CC2)c(c13)c(OC)c(c(F)c3)N(C4)C[C@H]([C@H]45)[NH2+]CCC5	252347	−4.990	Diaz et al.
N-des-methyl-clozapine	c1cccc(c1=2)=[NH+]c3c(cc(Cl)cc3)NC2N4CC[NH2+]CC4	4040	−7.481	Yu et al.
Olanzapine	Cc(c1)sc(c12)Nc3c(cccc3)N=C2N4CC[NH+](C)CC4	5830	−7.260	Diaz et al.
Perhexiline	C1CCCCC1C(C2CCCCC2)C[C@H]3CCCC[NH2+]3	2300	−7.820	Diaz et al.
Pimozide	c1cc(F)ccc1C(c2ccc(F)cc2)CCC[N@H+](CC3)CC[C@@H]3n4c(=O)[nH]c(c45)cccc5	28	−10.476	Diaz et al.
Propafenone	c1ccccc1CCC(=O)c2c(cccc2)OC[C@H](O)C[NH2+]CCC	1000	−8.322	Diaz et al.
Pyrilamine	n1ccccc1N(CC[NH+](C)C)Cc2ccc(cc2)OC	5000	−7.352	Diaz et al.
Quetiapine	OCCOCC[NH+](CC1)CCN1C2=Nc(cccc3)c3Sc(c24)cccc4	8040	−7.066	Diaz et al.
Quinidine	C=C[C@H]([C@H]12)C[N@H+](CC2)[C@H](C1)[C@@H](O)c3ccnc(c34)ccc(c4)OC	1310	−8.159	Diaz et al.

Ranolazine	c1ccc(C)c(c1C)NC(=O)CN2CC[NH+](CC2)C[C@@H](O)COc3c(OC)cccc3	21379	−6.477	Yu et al.
Risperidone	C1CCCn(c12)c(=O)c(c(n2)C)CC[N@H+](CC3)CC[C@@H]3c4noc(c45)cc(F)cc5	620	−8.610	Diaz et al.
Sertindole	N1CCN(C1=O)CC[N@H+](CC2)CC[C@@H]2c3cn(c(c34)ccc(Cl)c4)-c5ccc(F)cc5	34	−10.359	Yu et al.
Sotalol	CC(C)[NH2+]C[C@@H](O)c1ccc(cc1)NS(=O)(=O)C	24663	−6.391	Diaz et al.
Sparfloxacin	[O-]C(=O)c(c1=O)cn(C2CC2)c(c13)c(F)c(c(F)c3N)N(C[C@H]4C)C[C@H](C)[NH2+]4	18800	−6.555	Diaz et al.
Spironolactone	CC(=O)S[C@@H]1CC(=CC(=O)CC2)[C@@]2(C)[C@@H](CC3)[C@@H]1[C@H](CC4)[C@@]3(C)[C@]45CCC(=O)O5	45700	−6.020	Yu et al.
Terfenadine	CC(C)(C)c1ccc(cc1)[C@@H](O)CCC[N@H+](CC2)CC[C@@H]2C(O)(c3ccccc3)c4ccccc4	63	−9.987	Yu et al.
Terodiline	c1ccccc1C(C[C@@H](C)[NH2+]C(C)(C)C)c2ccccc2	860	−8.413	Yu et al.
Thioridazine	C[N@H+]1CCCC[C@H]1CCN2c(cccc3)c3Sc(c24)ccc(c4)SC	1065	−8.284	Yu et al.
Vanoxerine	c1cc(F)ccc1C(c2ccc(F)cc2)OCC[NH+](CC3)CCN3CCCc4ccccc4	60	−10.017	Yu et al.
Verapamil	COc(cc1)c(OC)cc1[C@](C#N)(C(C)C)CCC[N@@H+](C)CCc2cc(OC)c(cc2)OC	990	−8.328	Yu et al.

[a] ΔG is calculated by $\Delta G_{exp} = -RT \ln(1/K_i)$, where R is the gas constant and $T = 303.15$ K.

Supplementary Table S2. Calculated values of the absolute binding free energies (ΔG_{bind}) for 12 drugs.

Compound	ΔG_{exp} [a]	ΔG_{lig} [a]	ΔG_{comp} [a]	ΔG_{bind} [a]
Amiodarone	−9.03	−43.44	−50.51	−7.07
Amiodarone [b]			−60.14	−16.70
Astemizole	−11.93	−143.31	−166.82	−23.51
Chlorpromazine	−7.77	−46.54	−56.22	−9.68
Cisapride	−10.08	−49.20	−68.52	−19.32
Clofilium	−12.84	−37.82	−62.30	−24.48
Desipramine	−7.29	−47.33	−56.53	−9.20
Dofetilide	−11.47	−59.47	−78.21	−18.74
Grepafloxacin	−5.77	−112.87	−120.53	−7.66
Ketoconazole	−6.53	−51.27	−58.42	−7.15
Pimozide	−10.48	−57.85	−78.15	−20.30
Sotalol	−6.39	−55.80	−68.95	−13.15
Sotalol [b]			−67.59	−11.79
Verapamil	−8.33	−55.83	−70.43	−14.59

[a] The units for the binding free energies are kcal mol^{-1}.

[b] Results of the additional calculations.

Supplementary Table S3. Comparison of the binding free energies (ΔG_{pred}) predicted by our method and QikProp.

		Our method			QikProp		
Compound	ΔG_{exp} [a]	ΔG_{bind} [a]	ΔG_{pred} [a,b]	Error [c]	QPlogHERG	ΔG_{pred} [a,d]	Error [c]
Data for regression							
Amiodarone	−9.03	−16.70	−9.57	0.54	−6.23	−8.30	−0.73
Astemizole	−11.93	−23.51	−12.32	0.39	−8.12	−11.64	−0.29
Chlorpromazine	−7.77	−9.68	−6.74	−1.02	−6.11	−8.08	0.32
Cisapride	−10.08	−19.32	−10.63	0.55	−7.29	−10.17	0.09
Clofilium	−12.84	−24.48	−12.71	−0.14	N/A [e]	N/A [e]	N/A [e]
Desipramine	−7.29	−9.20	−6.55	−0.74	−6.08	−8.04	0.75
Dofetilide	−11.47	−18.74	−10.40	−1.07	−7.25	−10.11	−1.36
Grepafloxacin	−5.77	−7.66	−5.93	0.16	−3.36	−3.22	−2.56
Ketoconazole	−6.53	−7.15	−5.72	−0.81	−5.51	−7.03	0.49
Pimozide	−10.48	−20.30	−11.03	0.55	−8.09	−11.59	1.11
Sotalol	−6.39	−11.79	−7.59	1.20	−5.57	−7.12	0.73
Verapamil	−8.33	−14.59	−8.72	0.40	−7.07	−9.78	1.45
(RMSE)				0.71			1.12
Test data							
Bepridil	−9.39	−12.75	−7.98	−1.41	−6.72	−9.16	−0.23
E-4031	−10.94	−20.04	−10.92	−0.02	−7.06	−9.76	−1.17
Fluoxetine	−7.84	−13.29	−8.20	0.36	−6.67	−9.08	1.24
Ibutilide	−11.50	−21.42	−11.48	−0.02	−6.80	−9.30	−2.20
(RMSE)				0.73			1.40

[a] The units for the binding free energies are kcal mol^{-1}.

[b] $\Delta G_{\text{pred}} = (\Delta G_{\text{bind}} - 7.05) / 2.48$.

[c] Error = $\Delta G_{\text{exp}} - \Delta G_{\text{pred}}$.

[d] $\Delta G_{\text{pred}} = (\text{QPlogHERG} + 1.55) / 0.56$.

[e] Calculation of QPlogHERG failed for clofilium.

Amiodarone 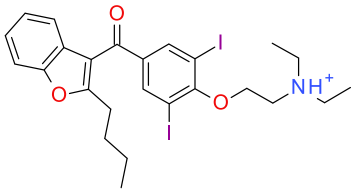	Astemizole 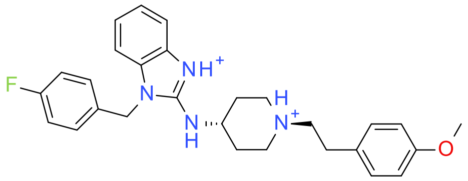
Chlorpromazine 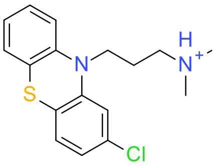	Cisapride 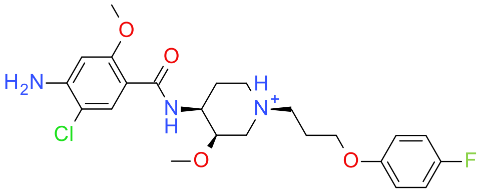
Clofilium 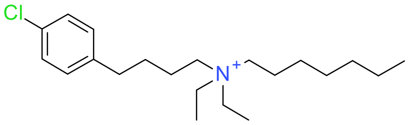	Desipramine 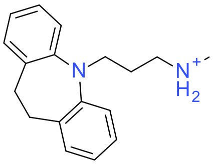
Dofetilide 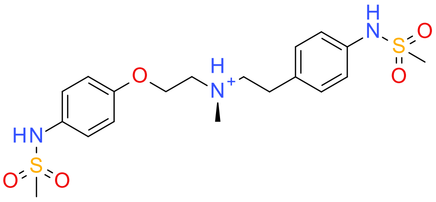	Grepafloxacin 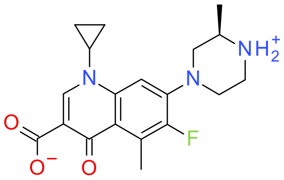
Ketoconazole 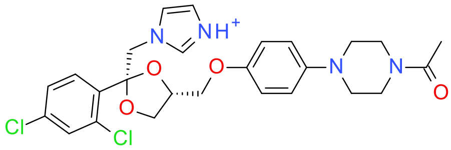	Pimozide 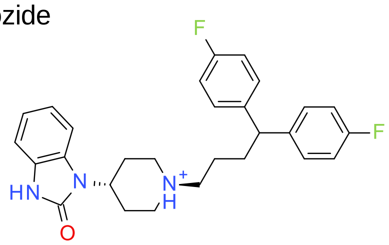
Sotalol 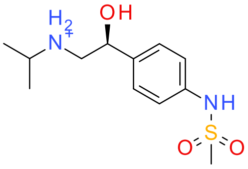	Verapamil 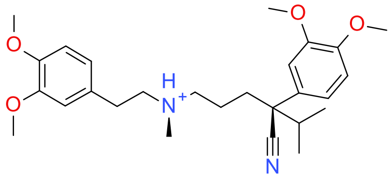

Supplementary Figure S1. Two-dimensional structures of the 12 compounds used for the absolute binding free-energy calculations.

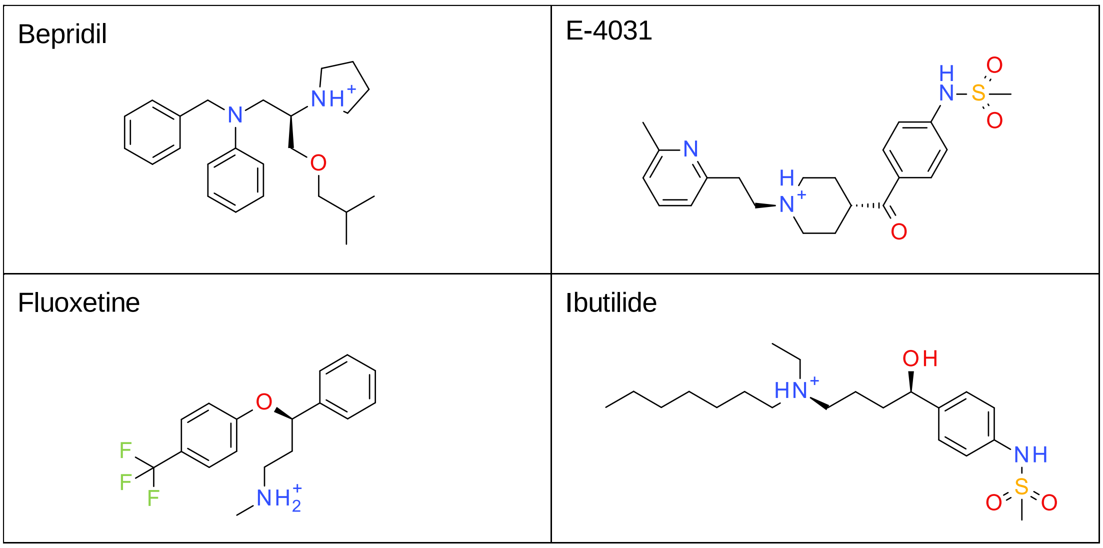

Supplementary Figure S2. Two-dimensional structures of the four compounds used as a test set.

a

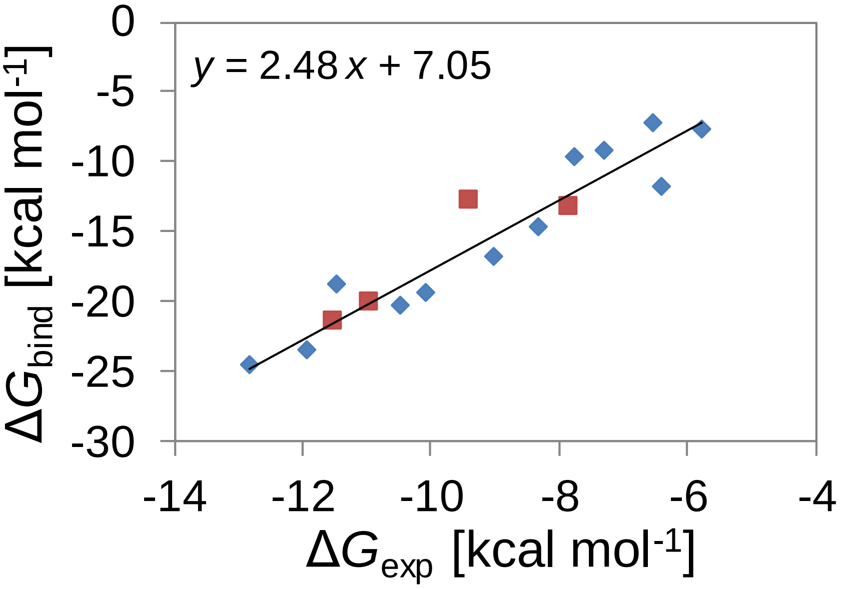

b

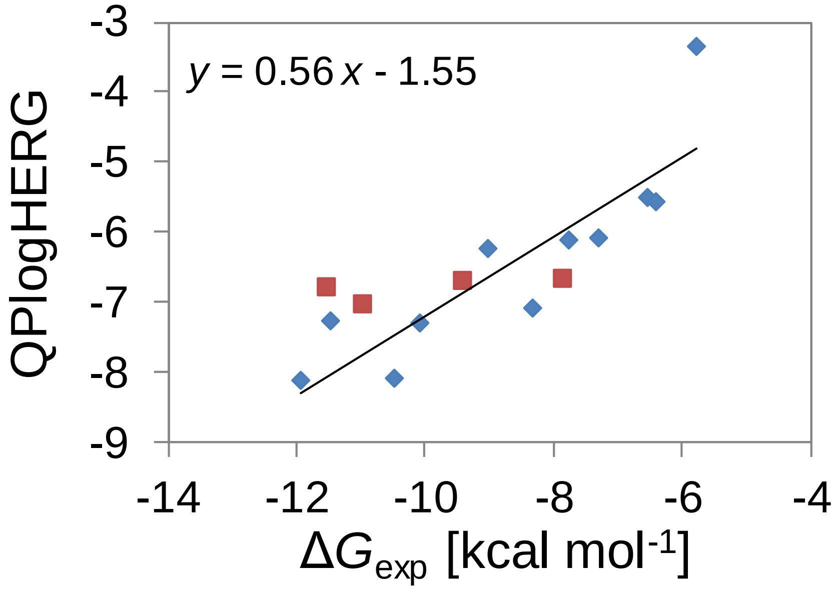

Supplementary Figure S3. Plots of the calculated values of the absolute binding free energy (ΔG_{bind}) (**a**) and the QPlogHERG descriptors (**b**) against the experimental binding free energy (ΔG_{exp}). Points colored blue represent the data of the 12 drugs for regression. Points colored red represent the data of the four drugs of the test data set. Solid lines represent the regression lines with a coefficient of determination (R^2) of 0.909 (**a**) and 0.765 (**b**). In each panel, the regression equation is shown, where x is ΔG_{exp} and y is ΔG_{bind}.

Supplementary References

1. Yu, Z., IJzerman, A. P. & Heitman, L. H. K_v11.1 (hERG)-induced cardiotoxicity: a molecular insight from a binding kinetics study of prototypical K_v11.1 (hERG) inhibitors. *Br. J. Pharmacol.* **172**, 940–955 (2015).
2. Diaz, G. J. *et al.* The [^{3}H]dofetilide binding assay is a predictive screening tool for hERG blockade and proarrhythmia: Comparison of intact cell and membrane preparations and effects of altering $[K^+]_o$. *J. Pharmacol. Toxicol. Methods* **50**, 187–199 (2004).
